# Canonical Wnt signaling is not required for *Tgfb3* expression in the basal medial edge epithelium during palatogenesis

**DOI:** 10.3389/fphys.2023.704406

**Published:** 2023-05-12

**Authors:** Ghazi-Abdullah Saroya, Erica Siismets, Max Hu, Christopher Panaretos, Adam Rice, Kurt Reynolds, Chengji J. Zhou, Vesa Kaartinen

**Affiliations:** ^1^ Department of Biologic and Materials Sciences, University of Michigan School of Dentistry, Ann Arbor, MI, United States; ^2^ Oral Health Sciences PhD Program, University of Michigan School of Dentistry, Ann Arbor, MI, United States; ^3^ College of Literature, Sciences and the Arts, University of Michigan, Ann Arbor, MI, United States; ^4^ School of Medicine, Institute for Pediatric Regenerative Medicine, Shriners Hospitals for Children-Northern California, University of California at Davis, Sacramento, CA, United States; ^5^ Department of Biochemistry and Molecular Medicine, School of Medicine, University of California at Davis, Sacramento, CA, United States

**Keywords:** beta-Catenin, TGF-beta3, canonical Wnt signal pathway, palatogenesis, cleft palate (CP)

## Abstract

The secondary palate forms from two lateral primordia called the palatal shelves which form a contact in the midline, become adherent at the fusing interface (medial edge epithelia, MEE) and subsequently fuse. The gene encoding transforming growth factor-ß3 (*Tgfb3*) is strongly and specifically expressed in MEE cells. Our previous study suggested that *Tgfb3* expression is controlled via upstream cis-regulatory elements in and around the neighboring *Ift43* gene. Another study suggested that the canonical Wnt signaling via ß-Catenin is responsible for the MEE-specific *Tgfb3* gene expression, since deletion of the *Ctnnb1* gene by a commonly used Keratin 14-Cre (*K14Cre*) mouse line almost completely abolished *Tgfb3* expression in the MEE resulting in cleft palate. Here, we wanted to analyze whether *Tcf/Lef* consensus binding sites located in the previously identified regions of the *Ift43* gene are responsible for the spatiotemporal control of *Tgfb3* expression during palatogenesis. We show that contrary to the previous report, deletion of the *Ctnnb1* gene in basal MEE cells by the *K14Cre* driver (the same *K14Cre* mouse line was used as in the previous study referenced above) does not affect the MEE-specific *Tgfb3* expression or TGFß3-dependent palatal epithelial fusion. All mutant embryos showed a lack of palatal rugae accompanied by other craniofacial defects, e.g., a narrow snout and a small upper lip, while only a small subset (<5%) of *Ctnnb1* mutants displayed a cleft palate. Moreover, the *K14Cre:Ctnnb1* embryos showed reduced levels and altered patterns of *Shh* expression. Our present data imply that epithelial ß-catenin may not be required for MEE-specific *Tgfb3* expression or palatal epithelial fusion.

## Introduction

Cleft palate, a common birth defect in humans, results from a failure in palatogenesis (Bush and Jiang, 2012; Lan et al., 2015). During this process, bilateral palatal primordia called palatal shelves grow out from the maxillary process of the first pharyngeal arch, elevating and fusing in the midline to form an intact secondary palate. Developing palatal shelves are mostly composed of the neural crest-derived mesenchyme and ectoderm-derived surface epithelium. The oral epithelium and developing palatal shelves are covered by a thin, one-cell layer thick tissue called the periderm. The periderm plays an important role in preventing aberrant fusions during oral development and must be eliminated at sites where fusion occurs, including epithelial tips of prefusion palatal shelves (Lan et al., 2015).

Palatal shelf growth and patterning are governed by complex reciprocal signaling processes between palatal mesenchymal and epithelial cells. Sonic hedgehog (*Shh*) expressed specifically in the oral epithelium in developing rugae is critical for controlling appropriate development of the size and shape of the palatal shelves, particularly in the anterior palate, by signaling to the adjacent mesenchyme (Bush and Jiang, 2012; Lane and Kaartinen, 2014).

During palatal shelf fusion, the palatal periderm must be eliminated before the underlying basal epithelial cells in the midline can become adherent (Hu et al., 2015; Richardson et al., 2017). How exactly this elimination takes place is currently controversial. It was initially thought that the periderm cells just slough off, undergo programmed cell death, or migrate toward the nasal and oral epithelia (Yoshida et al., 2012; Hu et al., 2015; Richardson et al., 2017). However, our recent studies suggest that the squamous periderm cells rapidly dedifferentiate into cuboidal epithelial cells (Saroya et al., 2023) incorporated into the midline seam, which soon disappears via mechanisms involving cell migration, convergence, and protrusion, allowing mesenchymal continuity necessary for appropriate fusion (Kim et al., 2015; Teng et al., 2022).

*Tgfb3* has been shown to be strongly and specifically expressed in both the basal (Fitzpatrick et al., 1990; Pelton et al., 1990) and peridermal (Lane et al., 2014) medial edge epithelium (MEE). *Tgfb3* expression is prerequisite for successful palatal fusion, since mice lacking *Tgfb3* display cleft palate with 100% penetrance (Kaartinen et al., 1995; Proetzel et al., 1995).

While signaling processes downstream of TGFβ3 are relatively well-understood (Bush and Jiang, 2012), very little is known about mechanisms governing *Tgfb3* expression in MEE cells of prefusion palatal shelves. It has been suggested that mutations in the *Foxe1* gene result in reduced *Tgfb3* expression in the MEE and that *Tgfb3* is a direct target of Foxe1 (Venza et al., 2011). Another study showed that deletion of the *Ctnnb1* gene by using the *K14Cre* mouse line, which induces recombination in the basal MEE cells (Lane et al., 2014; Saroya et al., 2023), completely eliminates *Tgfb3* expression in the MEE subsequently leading to a failure in palatal epithelial fusion (He et al., 2011).

To further characterize mechanisms targeting *Tgfb3* expression to the MEE, we used the *Tgfb3* BAC reporter lines (Lane et al., 2014) together with tissue-specific gene deletion and expression analyses. These assays suggested that *Tgfb3* expression in MEE cells recombined with *K14Cre* may not be controlled by canonical Wnt signaling. Instead, the *K14Cre:Ctnnb1* mutants displayed several previously reported phenotypes and expected alterations in *Shh* expression.

### Experimental procedures

#### Animal care

This study was carried out in accordance with the recommendations of the Guide for the Care and Use of Laboratory Animals of the National Institutes of Health. All the experiments involving animals described in this study were approved by the Animal Care and Use Committee of the University of Michigan-Ann Arbor (protocol number: PRO00004320).

#### Mouse lines

Generation of the #291 *Tgfb3* reporter line has been described earlier (Lane et al., 2014). Epithelium-specific *Ctnnb1* mutants with the *Tgfb3* reporter were obtained by crossing mice heterozygous for the floxed *Ctnnb1* and carrying the epithelial *K14Cre* driver (Andl et al., 2004) with homozygous floxed *Ctnnb1* (*Ctnnb1* ^*F/F*^) mice (Brault et al., 2001) carrying the #291 reporter (Lane et al., 2014). *Ctnnb1* ^*F*^ and *mTmG* reporter mice were obtained from the Jackson Laboratories. The mouse lines were maintained in a mixed (C57BL/6, 129SvJ and Black Swill) genetic background.

#### LacZ staining

Samples were briefly fixed in formaldehyde, then incubated in a solution of potassium ferrocyanide, ferricyanide, and X-Gal to develop color (Nagy et al., 2007). Samples were fixed overnight, imaged on the Leica M165FC stereomicroscope with DP73 and software.

#### DAPI dilactate staining

Samples were fixed overnight in formaldehyde, incubated in a 5 μg/mL solution of DAPI dilactate in PBS (Sandell et al., 2018) and imaged on fluorescence stereomicroscope as outlined above.

#### Histology and immunohistochemistry

Samples were dissected, fixed in 4% PFA, dehydrated in alcohol, and embedded in paraffin wax. 8 μm sections were stained with Hematoxylin and Eosin. Sections were viewed and documented using an Olympus BX51 microscope and an Olympus DP71 digital camera. For immunohistochemistry, tissues were fixed overnight in 4% paraformaldehyde, processed through sucrose gradient to OCT compound and embedded for cryo-sectioning. Sections were stained with β-Catenin (Cell Signaling #9587; dilution 1:200) and ZO1 (Invitrogen 33-9,100; dilution 1:200) primary antibodies and antibody binding was visualized with Alexafluor-488 and Alexafluor-594 -conjugated secondary antibodies (Life Technologies) on slides mounted with Prolong Diamond antifade/DAPI (ThermoFisher). Images were acquired by using a Leica DMi8 microscope controlled by Leica Application Suite X, 3.7.4.23463 software.

#### *In Situ* hybridization

ISH probes were made by performing *in vitro* RNA synthesis using digoxigenin-labeled ribonucleotides, RNA polymerase, and a linearized plasmid containing a cloned cDNA fragment of *Tgfb3* or *Shh* as previously described (Moorman et al., 2001). The staining procedure was performed on thin sections or whole tissue pieces by first treating with proteinase, briefly fixing with formaldehyde, and incubating the tissue with the digoxigenin-labeled RNA probe. An alkaline phosphatase-conjugated anti-digoxigenin antibody was applied to bind to the RNA probe, and the staining pattern was visualized by incubation with the BM purple chromogenic substrate solution.

#### qRT-PCR

Palatal shelves were dissected from embryos and total RNA was isolated with the RNeasy kit (Qiagen Cat # 74104). Reverse transcription was performed with the RT2 First Strand kit (Qiagen Cat # 330401). QPCR was performed with Taqman probes for *Tgfb3* (F-ccctggacaccaattactgc/R-tcaatataaagggggcgtaca), *Shh* (F-tccactgttctgtgaaagcag/R-gggacgtaagtccttcacca), and *Actb* (F-tgacaggatgcagaaggaga/R-cgctcaggaggagcaatg) as the housekeeping gene. 30mL reactions were quantified using Applied Biosystems ABI7300PCR and ViiA7 detection systems and software.

#### Micro-CT

Micro-CT scanning was performed with the µCT100 system (Scanco Medical, Bassersdorf, Switzerland) and scan settings: 12 µm voxel, 55 kVp, 109 μA, 0.5 mm AL filter, and 500 ms integration time. Image rendering and distance measurements were performed with the Microview software (Parallax Innovations). The landmarks used were published in Ho et al. (2015).

#### Statistical analysis

The Mann-Whitney non-parametric test was used to detect group differences for the qPCR results in Figure 2I (*n* = 10) and Figure 4C (*n* = 10). A multiple comparison-adjusted T-test was used to detect group differences for the micro-CT scanning measurements in Figure 3S (*n* = 3). An asterisk indicates a significant group difference with a false positive probability less than five percent (*p* < 0.05).

## Results

### *K14Cre:Ctnnb1* mutant mice do not show a clear reduction in *Tgfb3* expression

**FIGURE 1:**
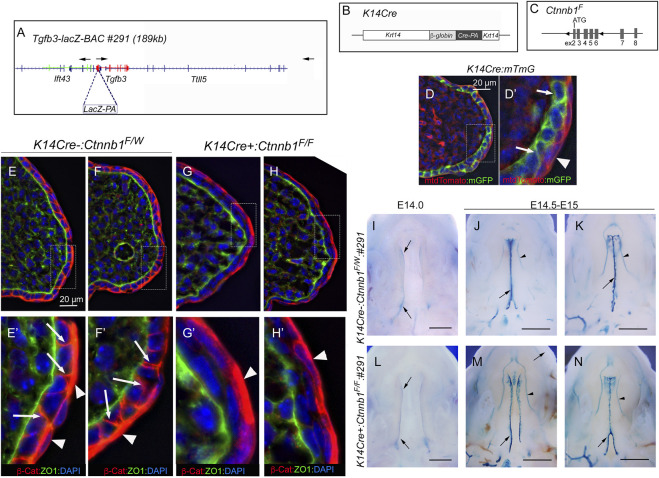
*Tgfb3* lacZ reporter mice do not reveal differences in the MEE between *K14Cre:Ctnnb1* mutant and control embryos. **(A)** Schematic presentation showing LacZ insertion into exon 1 of the *Tgfb3* gene in BAC RP24-299H18. Arrows show the position and orientation of the *Ift43*, *Tgfb3*, and *Ttll5* genes. **(B)** Schematic presentation of the *K14Cre* transgene. **(C)** Schematic presentation of the conditional *Ctnnb1* allele. LoxP sites surround exons 2-6 of the *Ctnnb1* gene. **(D)** *K14Cre*-induced recombination of the *mTmG* reporter cassette in pre-fusion palatal shelves. **(D′)** shows the high-power image of the field illustrated with a hatched box in **(D)**. Basal epithelial cells recombined with *K14Cre* show membrane-bound green fluorescence [white arrows in **(D′)**]. *K14Cre* does not recombine in the palatal periderm (red membrane-bound fluorescence highlighted with a white arrowhead). Counterstaining with DAPI (blue nuclear fluorescence). Scale bar, 20 µm. **(E–H)** Immunostaining demonstrates a loss of ß-Catenin in mutants [**(G, H)**; pre-fusion palatal shelves at E14.5; two separate embryos shown] when compared to control littermates [**(E, F)**; two separate embryos shown]. **(E′, F′, G′, H′)** show the high-power images of the fields illustrated with hatched boxes in **(E-H)**. White arrows in **(E′, F′)** point to adherent junctions of palatal basal epithelial cells. Similar staining cannot be seen in mutants **(G′, H′)**. White arrowheads in **(E′–H′)** point to palatal periderm cells that are not recombined by *K14Cre.* ZO1 immunostaining was used to highlight the basement membrane. Counterstaining with DAPI (blue nuclear fluorescence), ß-cat, ß-catenin. Scale bar, 20 µm. **(I–N)** Wholemount LacZ staining showing similar ß-galactosidase activity in the MEE of *K14Cre:Ctnnb1* mutant embryos **(L–N)** compared that of controls **(I–K)** at E14.0 **(I, J, L)** and E14.5-E15.0 **(J, K, M, N)**. Black arrows, positive reporter activity in the MEE; black arrowheads, reporter activity in blood vessels. Scale bars in **(I, L)**; 500 μm; **(J, K, M, N)**; 1 mm.

**FIGURE 2:**
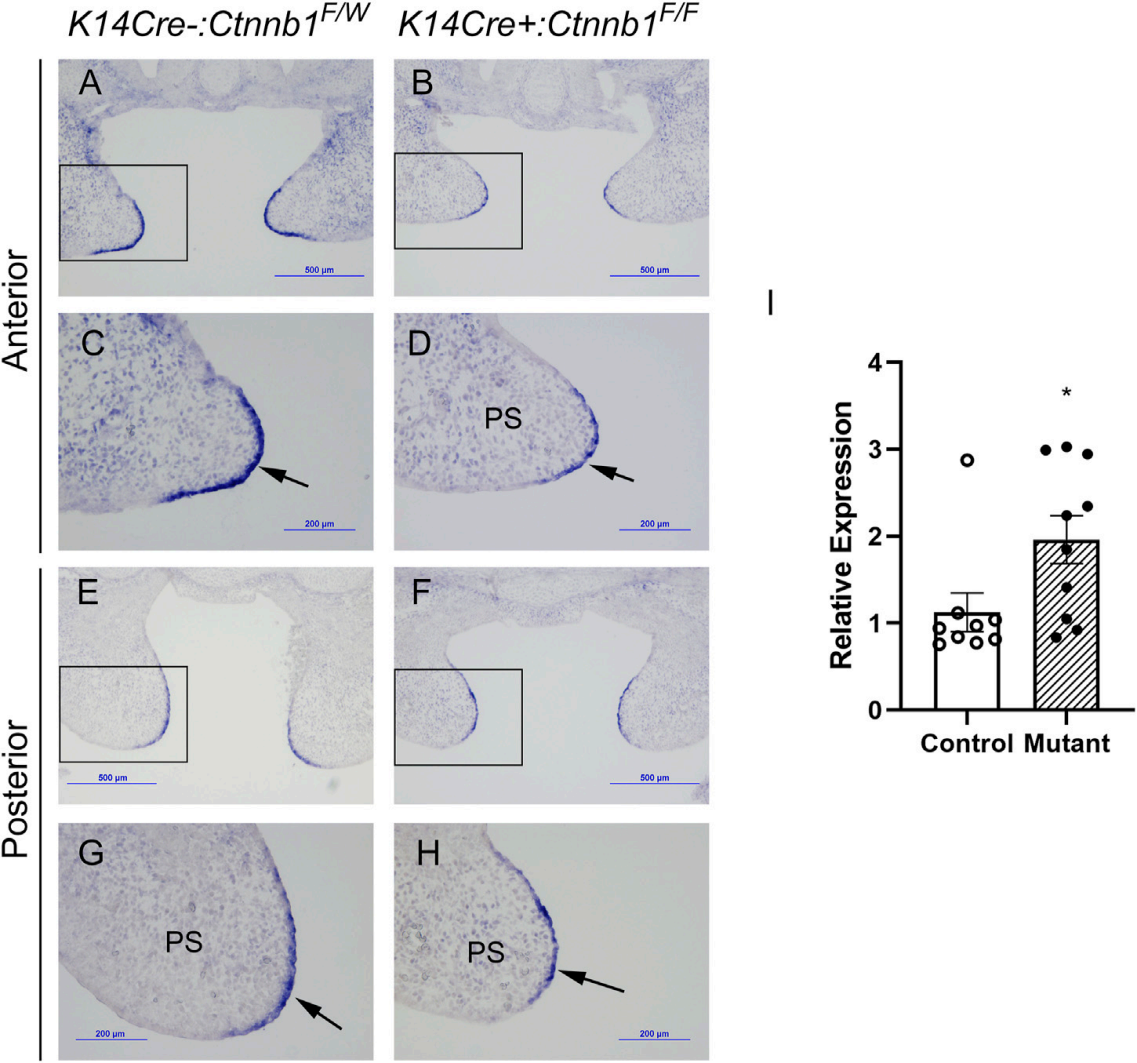
MEE-specific *Tgfb3* expression does not differ between control and *K14Cre:Ctnnb1* mutant embryos at E14.5. **(A–H)** *In situ* hybridization for *Tgfb3* on frontal sections of epithelium-specific *Ctnnb1* mutant **(B, D, F, H)** and control **(A, C, E, G)** embryos; Boxed area in **(A, B, E, F)**, are shown as high-power images in **(C, D, G, H)**, respectively. Black arrows point to the positive *Tgfb3* signal in the MEE. **(I)** Quantitation of *Tgfb3* mRNA using qRT-PCR. Control, open circles, and white column; mutant, closed circles and hatched column. Column height depicts the mean; error bar depicts the SEM. *Non-parametric test showed a group difference at the *p* < 0.05 significance level (*n* = 10).

It was previously reported that epithelial-specific *K14Cre:Ctnnb1* mutants show a nearly complete loss of *Tgfb3* expression in medial edge epithelial cells*,* which results in a failure in palatogenesis (He et al., 2011). To better understand the mechanisms by which canonical Wnt signaling regulates *Tgfb3* expression, we first tested whether epithelial-specific *K14Cre:Ctnnb1* mutants display reduced or absent *Tgfb3-lacZ* BAC reporter activity. *TheTgfb3-LacZ-BAC #291* (Figure 1) covers about 190-kb of mouse genomic sequence around the *Tgfb3* gene and recapitulates the specific pattern of the endogenous *Tgfb3* expression in the palatal epithelium (Lane et al., 2014). The commonly used *K14Cre* mouse line (Andl et al., 2004) induced efficient recombination of the mTmG reporter in the basal MEE cells, but not in periderm cells (Figure 1D). Similarly, immunostaining for β-Catenin implied that the *Ctnnb1* gene is inactivate in the basal MEE of *K14Cre:Ctnnb1* mutant embryos (Figure 1). Surprisingly, *theTgfb3-LacZ-BAC #291* displayed similar reporter activity in the MEE of the control and *K14Cre:Ctnnb1* mutant embryos (Figure 1). Next, we compared endogenous *Tgfb3* expression between control and *K14Cre:Ctnnb1* mutant mice to determine whether *Tgfb3* expression was unaffected in *Ctnnb1* mutants, or whether regulation of the reporter BAC differed from that of the endogenous *Tgfb3* gene (Figure 2). Similar to the results of the reporter assay, *in situ* hybridization analyses did not reveal notable differences in *Tgfb3* expression in the prefusion palatal epithelium between control and mutant samples. Prefusion palatal shelf tissues were harvested from control and mutant embryos and subjected to quantitative real-time PCR (*n* = 10). Results demonstrated that instead of being lost or dramatically reduced, overall *Tgfb3* mRNA levels were marginally increased in palatal tissues of *K14Cre:Ctnnb1* mutant embryos when compared to those of control littermates.

### *K14Cre:Ctnnb1* mutants show several craniofacial defects while the palatal epithelial fusion is not affected

**FIGURE 3:**
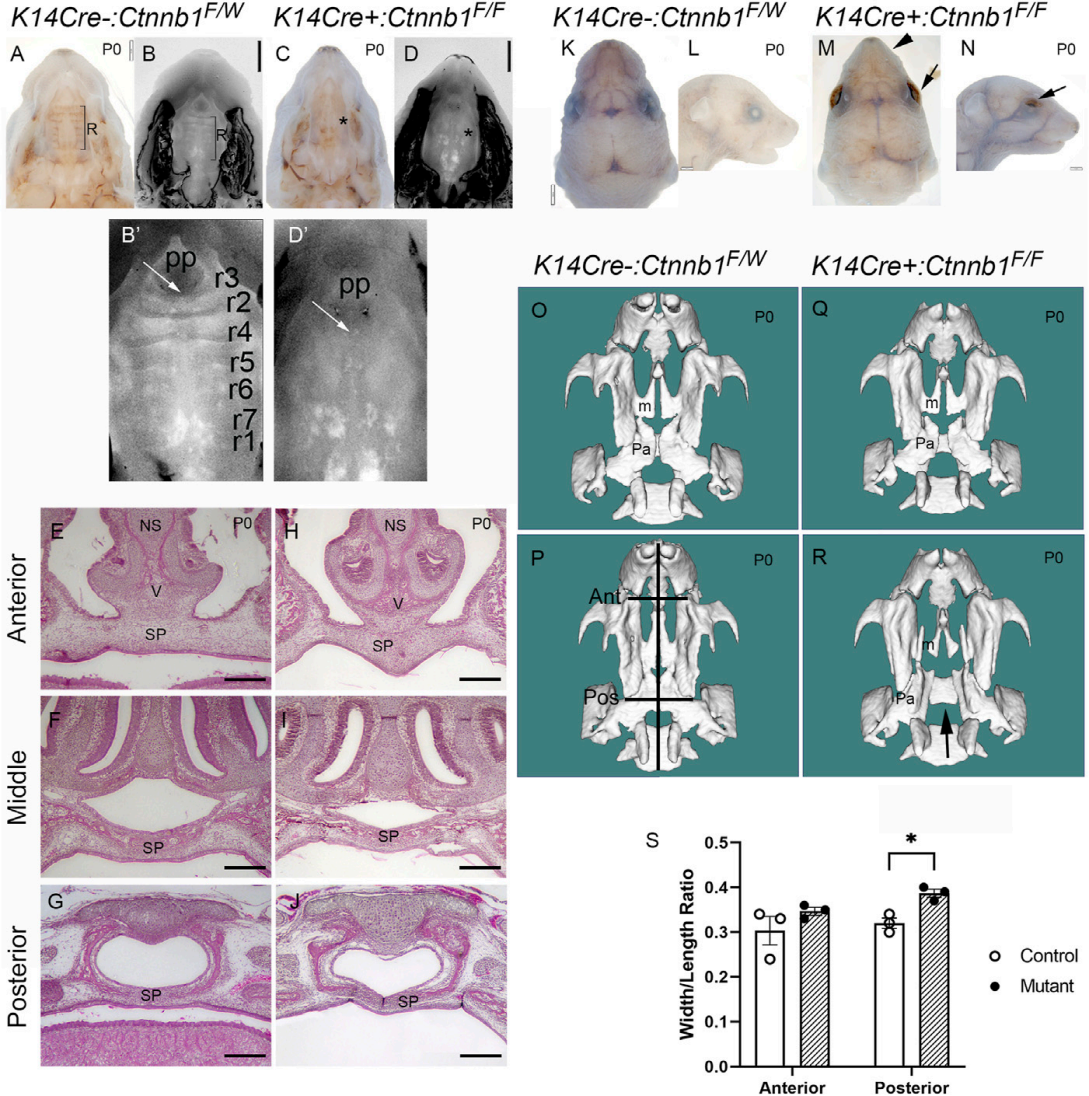
Craniofacial defects observed in *K14Cre:Ctnnb1* mutant embryos. **(A–D)** Bright field **(A, C)** and DAPI dilactate **(B, D)** images showing fused palate of control **(A, B)** and K14Cre:Ctnnb1 mutant newborn mice **(C, D)**. R in **(A, B)** depicts the rugae, * in **(C, D)** illustrates the absence of rugae. **(B′, D′)** magnified images shown in **(B,D)**. White arrows point to incisive foramen; pp primary palate. Rugae in **(B′)** have been numbered as shown in ref (Baek et al., 2011). **(E–J)** H&E staining of frontal sections showing fused palate of control **(E–G)** and *K14Cre:Ctnnb1* mutant **(H–J)** newborn mice (NS, nasal septum, SP, secondary Palate; V, Vomer) Scale bars, 500 µm. **(K–N)** Superior **(K, M)** and lateral **(L, N)** views of control **(K, L)** and *K14Cre:Ctnnb1* mutant **(M, N)** newborn mice. Black arrows **(M, N)** point to open eyelids, black arrowhead in M points to abnormal upper lip. **(O–R)** CT scan renderings of control **(O, P)** and *K14Cre:Ctnnb1* mutant **(R–S)** newborn mice. A mutant in **(R)** shows fused palate, while a mutant in **(S)** shows cleft palate (m, Maxillary bones; p, palatine bones; black arrows (S, T) point highlight the cleft palate. Black lines in P show the landmarks used for measurements in S; Ant, anterior; Pos, posterior).**(S)** Ratio of head length to anterior or posterior maxilla width. Control, open circles; mutant, closed circles. Column height depicts the mean; error bar depicts the SEM. *Multiple comparison-adjusted *T*-test showed a group difference at the *p* < 0.05 significance level (*n* = 3).

Next, we compared palatal and other craniofacial tissue tissues between control and mutant embryos. Consistent with the previous report (He et al., 2011), palatal rugae (Figures 3B’,D’) and whisker follicles (data not shown) were missing in *Ctnnb1* mutants. Mutants also displayed open eyelids and smaller upper lips compared to controls (Figures 3K–N). Despite these defects, most of the mutants showed seemingly normal closure of the secondary palate (Figures 3A–D). Serial sectioning of control and mutant tissues confirmed that palate fusion in *K14Cre:Ctnnb1* mutants was indistinguishable from that of controls throughout the anterior-posterior axis (Figures 3E–J). Among 64 *K14Cre:Ctnnb1* mutants analyzed, only three of them displayed cleft palate. Micro-CT analysis showed that regardless of the cleft palate phenotype, the maxilla of *K14Cre:Ctnnb1* mutant embryos appeared wider and shorter than those of controls (Figures 3O–S). In addition, this analysis demonstrated that even in the embryo which displayed a normally fused palate, the palatine bones were smaller than those in controls.

### Shh expression levels are reduced in *K14Cre:Ctnnb1* mutants

**FIGURE 4:**
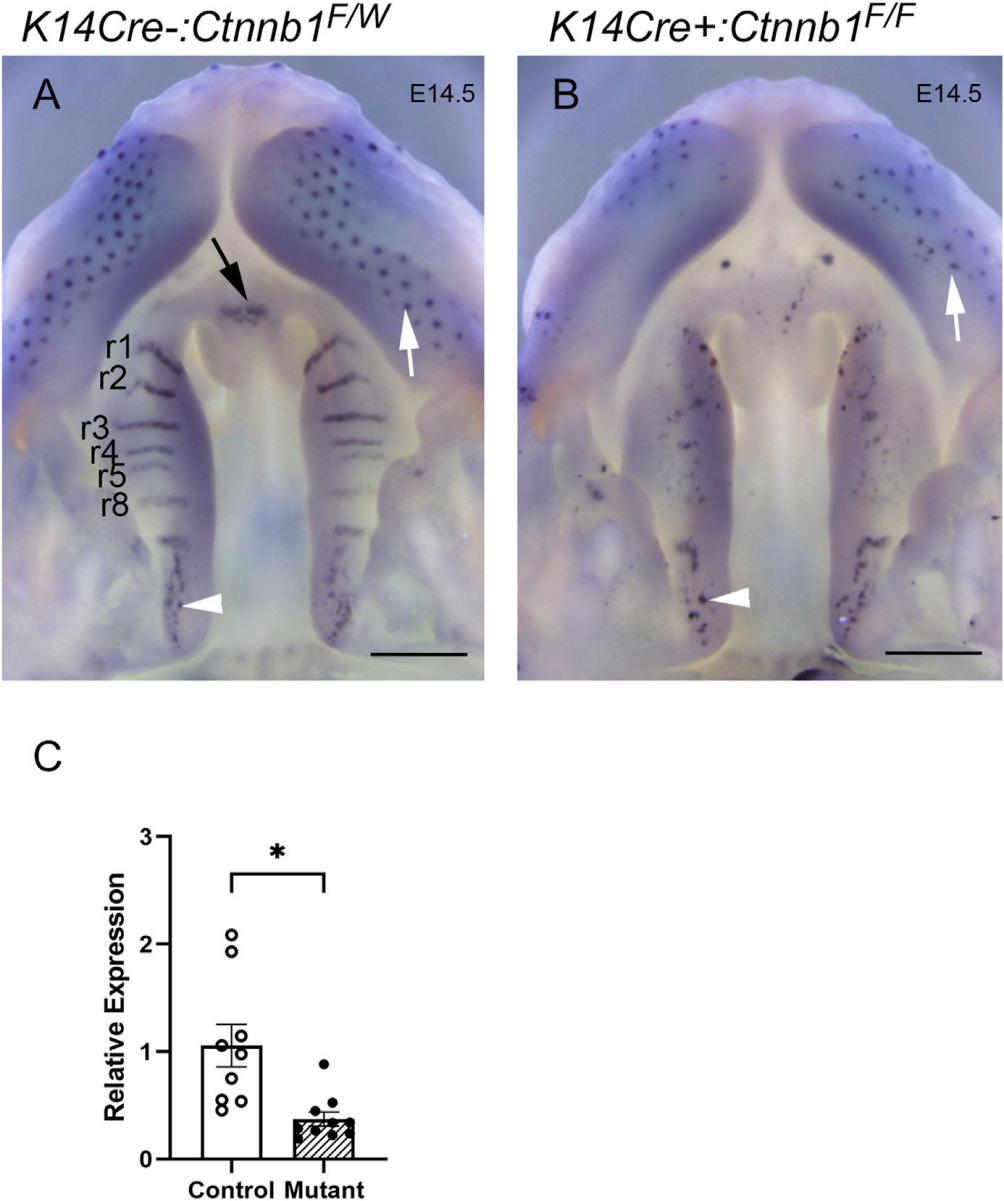
*Shh* expression in control and *K14Cre:Ctnnb1* mutant embryos at E14.5. Wholemount *in situ* hybridization for *Shh* in control **(A)** and *K14Cre:Ctnb1* mutant **(B)** embryos at E14.5. Black arrow **(A)** points to the positive signal in the nasal septum, white arrowheads **(A, B)** point to positive staining in taste buds in posterior palatal shelves, and white arrows **(A, B)** point to positive staining in hair follicles. Scale bars; 500 µm. **(C)** Quantitation of *Shh* mRNA using qRT-PCR. Control, open circles, and white column; mutant, closed circles and hatched column. Column height depicts the mean; error bar depicts the SEM. *Non-parametric test showed a group difference at the *p* < 0.05 significance level (*n* = 10).

*Ctnnb1* mutants displayed absence of rugae, of which development is dependent on Shh expression (Lee et al., 2011), and which specifically express Shh during normal palate development (Rice et al., 2006). Consistent with the lack of rugae, the *K14Cre*:*Ctnnb1* mutant embryos failed to show characteristic Shh-staining patterns on the oral side of the developing hard palate (Figure 4), while the posterior oral palate displayed Shh-positive taste buds comparable to those of controls. Additionally, aberrant *Shh*-positive spots reminiscent of taste buds could be seen in the mutant hard palate. Control embryos displayed positive *Shh* staining in the primary palate, while comparable staining could not be seen in mutants. Concordant with the *in situ* hybridization findings, q-PCR showed that *Shh* expression levels were reduced in mutant palatal tissues harvested from prefusion palatal shelves when compared to controls.

## Discussion

Palatogenesis is a complex developmental process involving reciprocal interactions between the mesenchyme and epithelium to govern tightly coordinated palatal shelf growth, elevation and fusion (Lan et al., 2015). Many previous studies have shown that *Tgfb3* is strongly and specifically expressed in the MEE of the prefusion palatal shelves and that it plays a critical role in successful palatal epithelial fusion (Fitzpatrick et al., 1990; Pelton et al., 1990; Kaartinen et al., 1995; Proetzel et al., 1995). However, very little is known about molecular mechanisms responsible for spatiotemporal control of *Tgfb3* expression during palatogenesis.

A previous study proposed that *K14Cre:Ctnnb1* mice lacking ß-Catenin in basal epithelial cells develop cleft palate and display complete absence of *Tgfb3* expression in the basal and periderm MEE. Using BAC transgenesis, we previously identified several distant cis-regulatory regions, which contributed the palate-specific *Tgfb3* expression (Lane et al., 2014). With the help of these previously generated reporter lines, we aimed to further explore the mechanisms by which canonical Wnt signaling regulates *Tgfb3* expression in MEE cells. Moreover, we aimed to clarify if indeed, the loss of *Ctnnb1* in the basal MEE could have influenced *Tgfb3* expression in the peridermal MEE, i.e., in cells in which *K14Cre* does not show the recombinase activity (Saroya et al., 2023). Our current results show that neither the *Tgfb3* reporter activity nor the endogenous *Tgfb3* expression levels were significantly affected in *K14Cre:Ctnnb1* mutants. Concordant with appropriate *Tgfb3* expression, palatal epithelial fusion occurred normally in most of the *K14Cre:Ctnnb1* mutants, while some (<5%) displayed cleft palate. However, the *K14Cre:Ctnnb1* mutant embryos consistently showed other phenotypes described in the previous study (He et al., 2011), e.g., a nearly complete absence of palatal rugae and notable reduction in *Shh* expression. Mutants also showed other craniofacial defects, including a narrow snout, small upper lip, open eyelids, and hind limb defects. Differences between the previous (He et al., 2011) and present studies could be attributed to variations in the genetic backgrounds of the mice used or discrepancies in the recombination efficiency within the *K14Cre* driver lines.

The role of Wnt signaling in rugae development and palate patterning is well-established (Lin et al., 2011), and many studies have examined interactions between Wnt and Hedgehog signaling processes in rugae patterning and palate morphogenesis (Lin et al., 2011; Sagai et al., 2017; Seo et al., 2018). Our studies agree with these earlier studies and confirm the critical role of canonical Wnt signaling in the regulation of Shh expression during palate formation and rugae patterning. Previous studies have shown that *Shh* expressed in the palatal epithelium acts on the mesenchyme, where it is required for appropriate growth and patterning of palatal shelves (Rice et al., 2004). The cleft palate observed with low penetrance in our *K14Cre:Ctnnb1* mutants may possibly be caused by altered *Shh* expression affecting maxillary and palatal shelf growth.

Our present data suggest that, at least in our outbred background, epithelial ß-catenin may not be required for MEE-specific *Tgfb3* expression and epithelial fusion of the secondary palate.

## Data Availability

The raw data supporting the conclusions of this article will be made available by the authors, without undue reservation.
